# The role of phosphorylated glucocorticoid receptor in mitochondrial functions and apoptotic signalling in brain tissue of stressed Wistar rats

**DOI:** 10.1016/j.biocel.2009.04.001

**Published:** 2009-11

**Authors:** Miroslav Adzic, Ana Djordjevic, Constantinos Demonacos, Marija Krstic-Demonacos, Marija B. Radojcic

**Affiliations:** a Laboratory of Molecular Biology and Endocrinology, VINCA Institute of Nuclear Sciences, P.O. Box-522-MBE090, 11001 Belgrade, Serbia; b School of Pharmacy Stopford building, University of Manchester, Oxford Road, Manchester, M13 9PT, England, UK; c Faculty of Life Sciences, Michael Smith Building, University of Manchester, Oxford Road, Manchester, M13 9PT, England, UK

**Keywords:** Glucocorticoid receptor, Phosphorylation, Mitochondria, Hippocampus, Prefrontal cortex

## Abstract

Mitochondrial dysfunction is increasingly recognized as a key component in compromised neuroendocrine stress response and, among other etiological causes, it may also involve action of glucocorticoid hormones. In the current study we followed glucocorticoid receptor and identified its mitochondrial phosphoisophorms in hippocampus and prefrontal brain cortex of Wistar male rats subjected to acute, chronic and combined neuroendocrine stresses. In both brain structures chronic social isolation caused marked increase in mitochondrial glucocorticoid receptor that was preferentially phosphorylated at serine 232 compared to serine 246 or serine 171. This increase corresponded with the decreased expression of mitochondrially encoded cytochrome oxidase subunits 1 and 3 in hippocampus, and with their increased expression in prefrontal brain cortex. Prefrontal brain cortex appeared to be more sensitive to chronic stress, since it exibited higher levels of mitochondrial Bax and cytoplasmic Bcl2 compared to hippocampus. Chronic stress also altered the response of both brain structures to subsequent acute stress according to the studied parameters. Therefore, prolonged social isolation may cause susceptibility to mitochondria triggered proapototic signalling, which at least in part may be mediated by the glucocorticoid receptor dependent mechanism.

## 1 Introduction

Glucocorticoid hormones (GCs) are secreted from adrenal glands upon activation of hypothalamic–pituitary–adrenal (HPA) axis and they regulate various physiological processes maintaining both basal and stress-induced homeostasis (De Kloet and Reul, 1987). The activity of HPA axis is controlled through inhibitory feedback mechanisms, involving upper brain structures, hippocampus (HIPPO) and prefrontal cortex (PFC) (Magarinos et al., 1987; Diorio et al., 1993). The GCs based feedback response at the cellular level is accomplished through the activity of glucocorticoid receptor (GR) protein (Sanchez et al., 1990). Upon ligand binding GR dissociates from the heterocomplex, translocates to the nucleus and regulates target gene expression.

Although GCs are the primary signal in activating GR transcriptional function, it may also be modulated by GR phosphorylation (Weigel and Moore, 2007). It was shown that rat GR is phosphorylated at threonine 171 (GRT171), serine 224 (GRS224), serine 232 (GRS232), or serine 246 (GRS246), which are all located in GR N-terminal domain (Krstic et al., 1997). Several protein kinases were shown to phosphorylate GR, each at the GR specific site, including a mitogen-activated protein kinase (MAPK) family member, c-Jun N-terminal kinase (JNK), glycogen synthase kinase-3 (GSK-3) and cyclin dependent kinases (CDKs) (Krstic et al., 1997; Ismaili and Garabedian, 2004; Kino et al., 2007). The phosphorylation by CDKs at GRS232 was shown to increase GR transcriptional activity, while phosphorylation by GSK-3 at GRT171 and phosphorylation by JNK at GRS246 exerts suppressive effect on GR transcriptional function (Ismaili and Garabedian, 2004; Rogatsky et al., 1998).

In addition to well-described molecular mechanism of GCs action these hormones can also directly or indirectly affect mitochondrial functions (Manoli et al., 2007). The presence of GR was reported within mitochondria of some brain cells (Koufali et al., 2003) and brain tissues (Moutsatsou et al., 2001). Considering crucial role of mitochondria in cell physiology, these organelles are among the first responders to various stresses that may affect cell homeostasis. The adaptive response to stress involves important changes in mitochondrial functions, enabling them to adjust bioenergetics, thermogenesis, oxidative and/or apoptotic responses (Manoli et al., 2007). Marked alterations in neuroendocrine responsiveness may be observed following disruption of the HPA axis by chronic stress (Armario et al., 1986; Hauger et al., 1990; Pitman et al., 1988) and are reflected on the mitochondrial processes, promoting respiratory chain dysfunction, decreasing ATP production, and causing mitochondrial structural abnormalities, apoptosis and cell death (Alesci et al., 2006).

The cellular energy state was shown to be regulated by GCs via GR that regulates both nuclear and mitochondrial genes involved in respiratory enzyme biosynthesis (Tsiriyotis et al., 1997). Mitochondrial GR was shown to control respiration and oxidative phosphorylation through transcriptional regulation of mitochondrial genes, the cytochrome oxidase 1 (COX 1) and cytochrome oxidase 3 (COX 3). Both COX 1 and COX 3 are catalytic subunits of cytochrome *c* oxidase, the last enzyme in the respiratory electron transport chain of mitochondria (Liang et al., 2006; Demonacos et al., 1996). Furthermore, in several cell lines, GR translocation to the mitochondria was shown to correlate with susceptibility to GCs induced apoptosis via mitochondrial pathway (Sionov et al., 2006).

In this study we investigated whether mitochondrial GR and/or its particular phosphoisoform may affect mitochondrial functions in a stress type dependent manner. Towards this end we followed GR, its phosphorylation and its cognate transcription function in mitochondria of hippocampus and prefrontal cortex of Wistar rats subjected to different stress models: acute, chronic and combined. The intracellular redistribution of Bcl2 family members that control initiation of apoptosis was also followed to assess the proapoptotic signals. Our working hypothesis is that mitochondrial GR and/or its particular phosphoisoform may affect mitochondrial functions. Here we document the presence of a specific GR phosphoisoform in mitochondria, which can play a role in modulation of expression of mitochondrial COX 1 and COX 3 genes and redistribution of members of Bcl2 family.

## 2 Materials and methods

### 2.1 Animals and treatment

The experiments were performed on adult (3 months old) Wistar male rats (body mass 330–400 g) housed four per standard size cage and offered food (commercial rat pellets) and water ad libitum. Light was kept on, between 7:00 a.m. and 7:00 p.m., and room temperature was kept at 20 ± 2 °C. All animal procedures were approved by the Ethical Committee for the Use of Laboratory Animals of the VINCA Institute of Nuclear Sciences, according to the guidelines of the EU registered Serbian Laboratory Animal Science Association (SLASA). The animals were divided into four groups: Group I consisted of unstressed animals (control group), in Group II animals were exposed to acute immobilization for 30 min, Group III animals were subjected to chronic isolation stress by housing them individually for 21 days, and Group IV was exposed to chronic isolation for 21 days followed by 30 min immobilization.

### 2.2 Corticosterone assay

Blood from each animal was collected at the time of sacrifice. Serum was prepared by 15 min centrifugation at 3000 rpm. The corticosterone (CORT) concentration was determined by using the OCTEIA Corticosterone EIA kit according to manufacturer's instructions (American Laboratory Products Co.). The absorbance at 450 nm was determined by microplate reader (Wallac, VICTOR^2^ 1420, PerkinElmer). CORT concentration (ng/ml) was determined using standard curve.

### 2.3 Preparation of brain tissues

All animals were sacrificed between 10:00 a.m. and –11:00 a.m., i.e. immediately after stress treatment, by rapid decapitation. The examined brain tissue, hippocampus and prefrontal cortex areas were removed and immediately frozen in liquid nitrogen until further preparation.

### 2.4 Preparation and characterization of cytoplasmic and mitochondrial extracts

Frozen tissues (HIPPO and PFC) were weighed and homogenized (1:2, w/v) in ice-cold 20 mM Tris–HCl (pH 7.2) buffer containing 10% glycerol, 50 mM NaCl, 1 mM EDTA, 1 mM EGTA, 2 mM DTT, and protease inhibitors (20 mM Na_2_MoO_4_, 0.15 mM spermine, 0.15 mM spermidine, 0.1 mM PMSF, 5 μg/ml antipain, 5 μg/ml leupeptin, 5 μg/ml aprotinin, 10 μg/ml trypsin inhibitor and 3 mM benzamidine) and phospatase inhibitors (20 mM β-glycerophosphate, 5 mM Na_4_P_2_O_7_ × 10H_2_O, 2 mM Na_3_VO_4_, 25 mM NaF) with 20 strokes of Potter-Elvehjem teflon-glass homogenizer. All operations were conducted at 0–4 °C. Samples were centrifuged 10 min at 2000 g to give a supernatant and a pellet (containing nuclear and cell debris and further processed for nuclear extracts). Supernatant was further centrifuged at 20,000 g for 30 min to obtain crude mitochondrial pellet. The resulting supernatant of this centrifugation was ultracentrifuged at 105,000 g for 1 h to obtain final supernatants used as cytoplasmic fraction. The crude mitochondrial pellet was washed (three times) in 0.5 ml of homogenization buffer and centrifuged at 20,000 g for 30 min. Mitochondrial pellets were then lysed in buffer containing 50 mM Tris–HCl (pH 7.4), 5% glycerol, 1 mM EDTA, 5 mM DTT, protease inhibitors and 0.05% Triton X-100 and incubated on ice for 1.5 h with frequent vortexing. Resulting fraction was used as final mitochondrial extract (Moutsatsou et al., 2001; Spencer et al., 2000; Trzeciak et al., 2004).

The purity of the cytoplasmic, mitochondrial and nuclear extracts were confirmed by Western blot analysis of the fractions, using antibodies directed against α-tubulin (T9026, Sigma), mHsp70 (MA3-028, Affinity Bioreagents) and NBS1 ([1D7] (MS-NBS10-PX1), GeneTex^®^,Inc.) for each compartment, respectively.

### 2.5 Western blot analysis

Protein concentration in the cytoplasmic and mitochondrial fraction was determined by method of Lowry (Lowry et al., 1951). The samples were mixed with denaturing buffer according to Laemmli (1970), boiled for 5 min at 100 °C, and 60 μg of proteins were subjected to electrophoresis on 7.5% or 12% sodium dodecyl sulfate-polyacrylamide gel (SDS-PAGE). After electrophoresis, proteins were transferred onto PVDF membrane (Immobilon-P membrane, Millipore) using a blot system (Transblot, BioRad). The membranes were incubated in blocking buffer: phosphate buffered saline (PBS) containing 5% milk for 1 h at room temperature, and thereafter probed overnight at 4 °C with specific primary antibodies diluted in PBS with 2.5% milk and 0.1% Tween 20. After washing three times in PBST, membranes were incubated with respective secondary antibody for 2 h at 4 °C, washed three times, soaked in enhanced chemiluminescence reagent (ECL, Pierce) and exposed to X-ray film. Protein molecular mass standards (PageRuler™ Plus Prestained Protein Ladder, Fermentas) were used for calibration. The following primary antibodies were used in mitochondrial fraction for detection of GR, and its phosphoisoforms, or mHsp70, which was used as loading control for mitochondrial extracts and did not vary with the treatment: BuGR2 (Affinity Bioreagents), phospho-GR (Ser211) antibody (Cell Signaling), and anti-mHsp70 antibody (Affinity Bioreagents). Anti-S246-P antibody was raised against phosphorylated rat GR peptide LLIDENLLpSPLAGEDDP and custom made by Sigma (Davies et al., 2008). Anti-T171-P antibody was raised against phosphorylated rat GR peptide STSATGCApTPTEKEFPK and custom made by Sigma. Rabbit polyclonal anti-β-actin (ab8227, Abcam) antibody was used as a loading control in the cytoplasmic compartment. For detection of Bax and Bcl2 proteins in mitochondrial and cytoplasmic fractions Santa Cruz Biotechnology (CA) antibodies were used. Blots were developed with ECL Rabbit IgG, HRP-linked whole antibody and ECL Mouse IgG, HRP-linked whole antibody (Amersham). Densitometry of protein bands on X-ray film was performed using Image J analysis PC software.

### 2.6 RNA extraction and reverse transcription

Total RNA, from HIPPO and PFC, was extracted using TRIzol^®^ Reagent (Invitrogen) according to manufacturer's instructions. Briefly, HIPPO and PFC tissues were weighed and homogenized in 1 ml TRIzol^®^ Reagent per 100 mg of tissue using Potter–Elvehjem teflon-glass homogenizer. Homogenates were then incubated at 30 °C for 5 min in order to completely dissociate nucleoprotein complexes. Sequentially, 0.2 ml of chloroform was added and the homogenate was shaken vigorously 15 s and incubated for 3 min at 30 °C. Samples were centrifuged at 12,000 g for 15 min at 4 °C. The aqueous phase, containing RNA, was mixed with 0.5 ml of isopropanol, incubated at 30 °C for 10 min and centrifuged at 12,000 g for 10 min at 4 °C. Resulting RNA pellet was resuspended in 75% ethanol, centrifuged (7500 g, 5 min, 4 °C), dried on air, and dissolved in 100 μl 0.1% DEPC water.

For the synthesis of cDNAs, a High-Capacity cDNA Reverse Transcription Kit (Applied Biosystems) was used according to manufacturer's instructions. Namely, 2 μg of total RNA was reverse transcribed using MultiScribe™ Reverse Transcriptase (50 U/μl) in the presence of 2 μl Random Primers, 0.8 μl 100 mM dNTP Mix, 1 μl of RNase Inhibitor and 10xRT Buffer in a final volume of 20 μl. The cDNAs were stored at −20 °C until use.

### 2.7 RTqPCR

The studied genes were amplified by SYBR green real-time PCR using Power SYBR Green PCR Master Mix (Applied Biosystems, Foster City, CA) in a 7500 Real-time PCR System (Applied Biosystems). Specific primers were used to selectively amplify cytochrome *c* oxidase subunit 1 (COX 1): F 5′-TCACAGTAGGGGGCCTAACA-3′, R 5′-GGCTTTTGCTCATGTGTCATT-3′ (X14848 Mitochondrial genome), cytochrome oxidase subunit 3 (COX 3): F 5′-TCTTCTTTGCCGGATTTTTC-3′, R 5′-ATGGTTTCGGTTGCCTTCTA-3′ (X14848 Mitochondrial genome) (Liang et al., 2006) and β-actin: F 5′-CTACAATGAGCTGCGTGTGGC-3′, R 5′- CAGGTCCAGACGCAGGATGGC-3′ (Figiel and Engele, 2000). cDNAs were amplified according to manufacturers protocols using the following conditions: hold 95 °C/10 min, denaturation 95 °C/15 s; annealing 60 °C/1 min, extension 60 °C/1 min and final extension 72 °C/5 min. Melt curve analyses were performed at the end of every experiment to confirm formation of a single PCR product. β-actin was used as an internal control to normalize the amount of RNA in each sample. Absolute values obtained for all samples were normalized to the β-actin signal included in each RTqPCR. As additional controls, PCR samples lacking only template were run for each set of reactions. Single peak melting profiles were obtained for all reactions and the size of the PCR products was confirmed by agarose gel electrophoresis. Each sample was run in triplicate and all experiments were repeated three times. Standard curves were used for each primer pair in order estimate the efficiency of amplification and *E* values were obtained from the slope of cycle threshold versus log concentration (Rutledge and Cote, 2003). The PCR efficiency was similar among all primers (0.96–0.99) and calculations of the relative amount of transcripts were performed using the ΔΔ*C*_T_ method (Livak and Schmittgen, 2001).

### 2.8 DNA fragmentation assay

Frozen samples of HIPPO and PFC were homogenized in lysis buffer containing 5 mM Tris–HCl pH 8.0, 20 mM EDTA and 0.5% Triton X-100. Homogenates were then centrifuged at 27,000 g for 20 min to separate intact chromatin in the pellets from fragmented DNA in the supernatant fractions. Pellets were resuspended in 0.5N perchloric acid and 5.5N perchloric acid was added to supernatant fractions to final concentration of 0.5 N. Samples were heated at 90 °C for 15 min and centrifuged at 1500 × *g* for 10 min to remove proteins. Supernatant fractions were reacted with diphenylamine for 16–20 h at room temperature and absorbance was measured at 600 nm. DNA fragmentation in the control samples is expressed as percent of total DNA appearing in the supernatant fraction. Treatment effects are reported as percent of control fragmentation (Bagchi et al., 1999).

### 2.9 Statistical analysis

Data are presented as mean ± SEM from three independent measurements of samples obtained from three separate groups of five animals (total number of animals 15 per experimental group). To determine statistical differences between stressed groups and control, as well as intergroup differences, we used one-way ANOVA test followed by the post hoc Tukey test. In order to simplify presentation of data all statistically significant differences are given as *p* < 0.05, including *p* < 0.01 and 0.001.

## 3 Results

### 3.1 Chronic stress lowers the level of corticosterone without impairing responsiveness to the subsequent acute stress

**Fig. 1:**
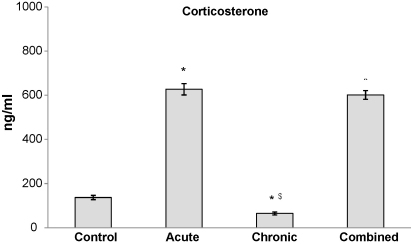
Serum corticosterone concentration (ng/ml) in control, acute, chronic and combined group of Wistar rats presented as mean ± SEM and measured individually. Statistically significant differences at the level of *p* < 0.05 also include *p* < 0.01 and 0.001 (*stress vs. control and ^$^chronic vs. combined).

In the control group of Wistar rats, the corticosterone (CORT) level was 137 ng/ml (Fig. 1), which was in the same range as reported by other authors (Merino et al., 2000; Wren et al., 2002). As expected, the acute (30 min) exposure to the high intensity physical–emotional–psychosocial stress, such as immobilization, resulted in a significant increase of serum CORT level to 626 ng/ml. On the contrary, chronic isolation for 21 days (low intensity but long-term psychosocial stress) led to significant decrease of CORT serum level to 65 ng/ml. This finding was in accordance with reports of other authors obtained on animals under similar conditions (Sanchez et al., 1998; Malkesman et al., 2006). When the chronically stressed animals were subsequently subjected to acute immobilization (i.e. combined stress), serum CORT increased to a similar level as that observed after acute stress (Fig. 1).

### 3.2 Chronic and combined stresses induce increase of mitochondrial level of total GR in the hippocampus and prefrontal cortex

**Fig. 2:**
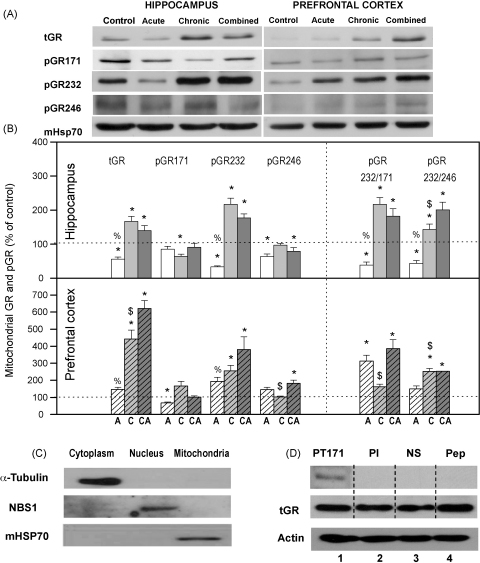
(A) Western blot experiments demonstrating the effects of acute immobilization chronic isolation and combined stress on the level of total glucocorticoid receptor (tGR) and its phosphoisoforms in mitochondria of hippocampus and prefrontal cortex. Mitochondrial lysates were resolved by SDS-PAGE and probed with antibodies against tGR, pGR171, pGR232, pGR246 or mHsp70 as a loading control. (B) Immunoreactivities of mitochondrial tGR, pGR171, pGR232, pGR246 (normalized to mHsp70) in hippocampus and prefrontal cortex. The ratios of pGR232/171 and pGR232/246 (normalized to GR and mHsp70) are expressed as mean ± SEM and presented as a percent of control (as described under Section 2). Asterisk indicates significant differences between treated groups: acute (A), chronic (C) and combined (CA) obtained from one-way ANOVA followed by Tukey post hoc test (*p < 0.05, stress vs. control; ^%^*p* < 0.05, acute vs. combined; ^$^*p* < 0.05 chronic vs. combined). (C) The purity of subcellular fractions was assayed using specific antibodies against α-tubulin for cytoplasmic, NBS1 for nuclear, and mHsp70 for mitochondrial fraction. (D) Western blot of GR probed with antibody specific for its phosphorylated T171 isoform in the absence (lane 1) or presence (lane 4, PEP) of specific peptide used as antigen for this antibody. Membrane strips were also incubated with preimmune serum (lane 2, PI) or with non-specific IgG (lane 3, NS) (top panel). Blot was stripped of primary antibodies and probed with GR specific antibody (middle panel). Lower part of this blot was probed with actin antibody.

Decreased level of total GR (tGR) was observed in mitochondria of HIPPO following acute stress (Fig. 2A and B) while in animals subjected to chronic stress mitochondrial tGR was significantly increased. When chronically stressed animals were subsequently subjected to acute stress (i.e. combined stress), the level of mitochondrial tGR was also significantly increased compared to control and slightly decreased compared to chronic stress. In PFC, the mitochondrial tGR was significantly elevated by chronic and combined stresses, while it was unchanged under the acute stress (Fig. 2A and B).

### 3.3 Increased mitochondrial GR found under chronic and combined stresses is predominantly phosphorylated at serine 232

Since phosphorylation has been proposed to influence the GR activity (Krstic et al., 1997; Davies et al., 2008) in the next set of experiments we analyzed phosphorylation pattern of the mitochondrial GR. We used antibodies specific for the GR isoforms phosphorylated at T171, S232 and S246 and our results indicated presence of all three GR isoforms in the mitochondria of HIPPO and PFC of untreated animals (Fig. 2A). Specificity of pT171 antibody custom made by Sigma is shown in Fig. 2D. Specific GR band was detected with pT171 antibody and not with preimmune serum, non-specific antibody or pT171 antibody preincubated with the specific peptide. Specificity of pS246 antibody has previously been reported by our group (Davies et al., 2008) whereas specificity of pS211 (S232 in the rat nomenclature) was indicated by Sigma and by Ismaili and Garabedian (2004).

In the next set of experiments we detected that the pattern of mitochondrial GR phosphoisoforms was changed by different stress conditions. For example, the mitochondrial GRT171 phosphoisoform was downregulated in HIPPO by chronic stress while in PFC, it was downregulated by the acute stress (Fig. 2A and B). The mitochondrial GRS232 phosphoisoform in HIPPO was markedly decreased by acute stress, but contrary to that it was increased by chronic and combined stresses (Fig. 2A and B). In PFC, GRS232 phosphoisoform was increased by all three types of stresses. The mitochondrial GRS246 phosphoisoform in HIPPO was significantly decreased under acute and combined stresses (i.e. high CORT), but unchanged by chronic stress, while in PFC it was affected only by combined stress (Fig. 2A and B).

**Fig. 3:**
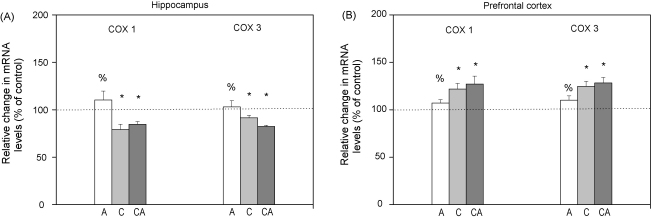
Real time RTqPCR analysis of relative changes in cytochrome oxidase subunit1 (COX 1) and cytochrome oxidase subunit 3 (COX 3) mRNA levels under acute (A), chronic (C) and combined stress (CA) in rat hippocampus and prefrontal cortex. Calculations were performed using ΔΔ*C*_T_ method. Data are presented as mean ± SEM as a percent of control and statistical analyses was performed using one-way ANOVA followed by Tukey post hoc test (*p < 0.05, stress vs. control; ^%^*p* < 0.05, acute vs. combined; ^$^*p* < 0.05 chronic vs. combined).

Although a direct comparison of GR phosphoisoform recruitment is confounded by potential differences in the avidity of the phospho-antibodies, similarly to Chen et al. (2008) we used the ratio of different GR phosphoisoforms to speculate about their mechanistic implications in regulation of COX genes expression (Figs.2 and 3). The ratio values above one hundred percent indicated the dominance of GRS232 phosphoisoform, while values below one hundred percent, the prevalence of GRT171 and GRS246 phosphoisoforms. This approach indicated that in HIPPO, phosphorylation at S232 of mitochondrial GR was diminished compared to phosphorylation at T171 and S246 under acute stress (Fig. 2B). On the contrary, phosphorylation at GRS232 was predominant compared to that at T171 and S246 under chronic and combined stresses. Furthermore, the increase in mitochondrial tGR in HIPPO under chronic and combined stresses correlated well with its elevated phosphorylation at S232 (Fig. 2A and B).

The analyses of GR phosphoisoform ratios in PFC indicated that phosphorylation at S232 prevailed that at T171 under acute and combined stresses (i.e. high CORT) and prevailed that at S246 under chronic and combined stresses. Similarly like in HIPPO, the increase in mitochondrial tGR in PFC correlated with elevated GR phosphorylation at S232 (Fig. 2A and B).

### 3.4 Chronic stress differentially regulates the expression of COX 1 and COX 3 genes in hippocampus and prefrontal cortex

Considering the fact that COX 1 and COX 3 are encoded by the mitochondrial genome (Liang et al., 2006) and that both genes contain GREs (Demonacos et al., 1995; Demonacos et al., 1996) we exploited real time RTqPCR technique to evaluate the potential correlation of mitochondrial GR phosphorylation pattern with the expression of COX 1 and COX 3 genes in HIPPO and PFC of stressed Wistar rats. As shown in Fig. 3A, acute stress did not affect expression of COX 1 and COX 3 genes in the HIPPO, while it was significantly decreased under chronic and combined stresses. In contrast to HIPPO, the expression of COX 1 and COX 3 genes in PFC was significantly increased by chronic and combined stresses (Fig. 3B). Under acute stress, their expression was unchanged compared to control.

### 3.5 Chronic and combined stresses modulate intracellular distribution of Bcl2 family members

**Fig. 4:**
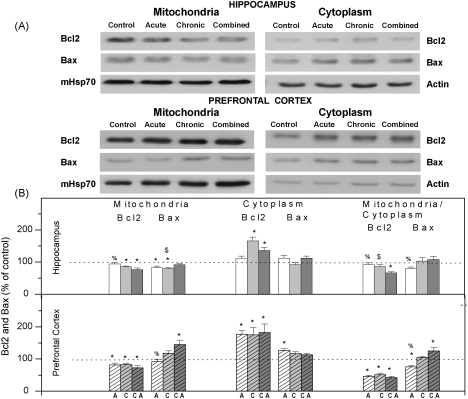
(A) Western blot experiment demonstrating the effect of stress on mitochondrial and cytoplasmic Bcl2 and Bax levels in rat hippocampus and prefrontal cortex. (B) Immunorectivities of Bcl2 and Bax (normalized to mHsp70) in mitochondrial extracts and cytoplasm (normalized to β-actin) of hippocampus or prefrontal cortex under acute (A), chronic (C) and combined (CA) stress and the ratio of mitochondrial to cytoplasmic Bcl2 and Bax are given as mean ± SEM, as a percent of control (as described under Section 2). Asterisk indicates significant differences obtained from one-way ANOVA followed by Tukey post hoc test (*p < 0.05, stress vs. control; ^%^*p* < 0.05, acute vs. combined; ^$^*p* < 0.05 chronic vs. combined).

In order to analyse potential relation between mitochondrial GR phosphoisoforms and mitochondria triggered apoptosis we followed the intracellular distribution of proapoptotic and antiapoptotic members of Bcl2 family, namely Bax and Bcl2, in the cytoplasm and mitochondria of HIPPO and PFC under stress conditions. As shown in Fig. 4A and B, the level of Bcl2 protein in the mitochondrial extracts of HIPPO was decreased under chronic and combined stresses. Parallel increase of Bcl2 in the cytoplasm of HIPPO under the same stress conditions was observed. The level of mitochondrial Bax protein was decreased by the acute and chronic stresses, while its level in the cytoplasm was not affected by any of the stress conditions. In PFC, we observed decrease of mitochondrial Bcl2 and in parallel its increase in the cytoplasm under all stress conditions (Fig. 4A and B). The mitochondrial Bax was only significantly increased by combined stress, while in cytoplasm it was increased only under acute stress. When the mitochondrial/cytoplasmic ratios of Bcl2 to Bax were analysed (Fig. 4B) we observed decrease of Bcl2 in the cytoplasm and slight increase in mitochondrial Bax under the combined stress in HIPPO. Moreover in PFC, the ratio of mitochondrial/cytoplasmic Bcl2 to Bax indicated translocation of Bcl2 from the mitochondria to the cytoplasm by all three stress types and elevation of mitochondrial Bax under the combined stress (Fig. 4A and B).

### 3.6 Chronic stress increases the level of DNA fragments in hippocampus and prefrontal cortex

**Fig. 5:**
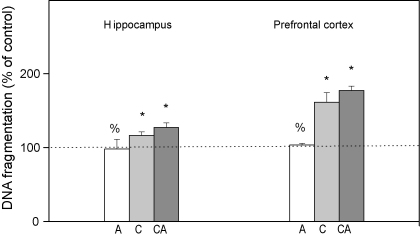
Measurement of DNA fragments with the diphenylamine (DPA) colorimetric assay. Results are presented as a percent of control fragmentation following formula % of fragments = (OD_600 nm_T/OD_600 nm_(T + B)) × 100, *T* = fragmented DNA, *B* = intact DNA obtained from rats exposed to acute (A), chronic (C) and combined (CA) stress. Statistical analyses was performed using One-way ANOVA followed by Tukey post hoc test (*p < 0.05, stress vs. control; ^%^*p* < 0.05, acute vs. combined; ^$^*p* < 0.05 chronic vs. combined).

In another set of experiments we calculated the fraction of fragmented DNA out of total DNA isolated from HIPPO and PFC of stressed Wistar animals (Filipkowski et al., 1994). As shown in Fig. 5, the increased levels of DNA fragments in both brain structures were observed under chronic and combined stresses. In response to acute stress, the level of DNA fragments was unchanged in the both observed brain structures (Fig. 5A and B).

## 4 Discussion

In the present study we have documented alterations in the basic (control) level of mitochondrial glucocorticoid receptor and identified changes in its phosphorylation pattern in hippocampus and prefrontal cortex of Wistar male rats subjected to different types of neuroendocrine stress.

Thus, in animals subjected to acute stress (30 min immobilization) which exhibited marked increase in serum corticosterone (CORT), we found that the total GR level in mitochondria of HIPPO was decreased (Fig. 2, tGR). This decrease occurred simultaneously with a prominent translocation of cytoplasmic GR to the nucleus of HIPPO (Adzic et al., under review). Thus, it seemed that the hippocampal response to acute stress involved redirection of mitochondrial GR to other cellular compartments of HIPPO. Nevertheless, in spite of marked GR translocation from cytoplasm to the nucleus in PFC (Adzic et al., under review), the level of mitochondrial GR in PFC remained unaltered by acute stress. Therefore, our findings point out differential steroid sensitivity of the mechanisms by which GR is transported to or from the mitochondria in the two brain tissues. In addition to that, the analysis of mitochondrial GR phosphorylation in HIPPO and PFC indicated that the basic (control) pattern of GR phosphorylation (regarding all three epitopes: GRT171, GRS246 and GRS232, Fig. 2) was differentially modified by acute stress. Thus, in HIPPO we observed prevalence of GRT171 and GRS246 over GRS232 phosphoisoform, while in PFC the GRS232 phosphoisoform was dominant (Fig. 2, pGRs ratios). Since GR phosphorylation at S232 epitope was previously shown to increase GR transcriptional activity, while that at T171 and S246 epitopes exhibited suppressive effects (Ismaili and Garabedian, 2004), our findings suggested that acute stress may induce tissue specific alterations in the transcriptional activity of mitochondrial GR in brain. To test this presumption we analysed the expression of the two mitochondrial encoded genes, COX 1 and COX 3, known to be regulated by GCs via GR (Demonacos et al., 1996). This analysis indicated that acute immobilization did not affect COX 1 and COX 3 gene expression, suggesting that respiration parameters were not altered in either of the two brain structures (Fig. 3). Nevertheless, according to previous reports of Sionov et al. (2006), elevation in GCs may induce apoptosis via GR translocation to the mitochondria. In order to follow the initial phase of this process we analyzed relocation of cytoplasmic pool of both Bcl2 and Bax to mitochondria (Cao et al., 2001; Gajkowska et al., 2001). We observed Bcl2 translocation from mitochondria to the cytoplasm in both tissues, while cytoplasmic Bax was elevated only in PFC (Fig. 4). Also, in either of the brain structures, we did not observe DNA fragmentation, which is an indication of cellular apoptosis (Kerr et al., 1995; Li et al., 1995a,b) (Fig. 5). Taken together our results showed somewhat higher susceptibility of PFC to initiation of mitochondrial proapoptotic signalling. Also, since our analysis was performed immediately after acute stress, this result did not preclude the possibility of apoptosis at later time points.

The second type of stress we exploited in this study was a long-term social isolation. In spite of decreased CORT levels in blood serum of these animals (Sanchez et al., 1998; Malkesman et al., 2006) the level of mitochondrial GR was increased in both brain structures. Thus, it seemed that GR accumulation in brain mitochondria may be governed by a CORT independent mechanism. This mitochondrial GR was prevalently phosphorylated at S232 compared to GRT171 and GRS246 in both tissues (Fig. 2), indicating that chronic stress may also alter transcriptional activity of mitochondrial GR. Furthermore, elevated level of mitochondrial GRS232 coincided with decreased expression of COX 1 and COX 3 genes in HIPPO and their increased expression in PFC (Fig. 3). The differential regulation of COX 1 and COX 3 genes by the same GR phosphoisoform (GRS232) in the two tissues may reflect diversity and complexity of positive or negative GR transcriptional activity due to tissue specific cofactors, as previously observed by Kino et al. (2007). These differences are likely to affect the functionality of the cytochrome *c* oxidase enzyme, and hence energy production in these tissues, in an opposite manner. Bearing in mind that decreased COX mRNA levels may influence cytochrome *c* oxidase activity leading to mitochondrial malfunctions (Gao et al., 2007) our results point out that chronic stress may compromise mitochondrial functions in HIPPO. Elevated COX 1 and COX 3 mRNA in PFC may be connected with an increased cytochrome *c* oxidase activity (Liang et al., 2006), indicating greater energy demands of PFC in response to chronic stress. Chronic stress also caused redistribution of Bcl2 family members and elevated level of DNA fragments in both tissues, again, with more prominent changes in PFC (Figs. 4 and 5). Since, mitochondrial localisation of GR is necessary for glucocorticoid-induced apoptosis (Sionov et al., 2006), our results indicate that GRS232 isoform may be responsible for this processes. Namely, this isoform of GR, through regulation of respiration, may alter mitochondrial functions favouring initiation of proapoptotic signaling in both tissues under chronic stress.

Finally, we used ‘the combined stress model’ (isolation plus immobilization) to investigate potentially maladaptive effects of chronic stress, defined as irreversible alterations in studied parameters. Since, CORT levels in these animals were again increased, we concluded that in spite of the previous chronic stress exposure, HPA axis responsiveness remained intact regarding this parameter. However, the additional acute stress did not restore the level of mitochondrial GR which remained increased in both HIPPO and PFC. The analyses of its phosphorylation status again showed the prevalence of GRS232 isoform in both brain structures (Fig. 2). This GR phosphorylation pattern was similar to the one found under chronic stress, i.e. it was not reversed by subsequent acute stress (Fig. 2). In accordance with this result, the mRNA levels of COX 1 and COX 3 genes were not restored by the additional acute stress neither in HIPPO nor in PFC (Fig. 3). Combined stress also caused redistribution of Bcl2 family members (Fig. 4) and increased level of DNA fragments in both brain structures (Fig. 5). Taken together, these findings suggested that the increase in mitochondrial GRS232 isoform under combined stress may again be responsible for these processes since they were not reversed.

In summary, the presented data provide evidence that chronic stress leads to accumulation of GR in mitochondria of hippocampus and prefrontal cortex of Wistar rats. The mitochondrial GR is primarily phosphorylated at S232 compared to the two other GR phospho epitopes T171 and S246, and its increase correlated with altered expression of mitochondrial COX 1 and COX 3 genes. Although COX genes are regulated in an opposite manner that may presumably lead to divergent effects in the respiratory parameters of the two brain structures, both structures exhibited similar pattern of proapoptotic signalling via relocation of Bcl2 family members. These mitochondrial characteristics of GR could not be reversed by subsequent acute stress, unlike the reversibility of nuclear GR characteristics set by chronic stress (Adzic et al. under review). Therefore it may be concluded that chronic stress directs transcriptionally active GR to the mitochondria of Wistar rat brain, and simultaneously renders them more susceptibile to proapoptotic signalling. Taken together our data support findings of other authors (Sionov et al., 2006; Manoli et al., 2007; Psarra and Sekeris, 2008) that GR triggered signalling at the level of brain mitochondria may be a key modulator of stress response.
